# Vertical Distribution of Oviposition and Temporal Segregation of Arbovirus Vector Mosquitoes (Diptera: Culicidae) in a Fragment of the Atlantic Forest, State of Rio de Janeiro, Brazil

**DOI:** 10.3390/tropicalmed8050256

**Published:** 2023-04-29

**Authors:** Rayane Dias, Cecilia Ferreira de Mello, Gabriel Silva Santos, Ana Laura Carbajal-de-la-Fuente, Jeronimo Alencar

**Affiliations:** 1 Laboratório Diptera, Instituto Oswaldo Cruz (FIOCRUZ), Avenida Brasil 4365, Manguinhos, Rio de Janeiro 21040-360, RJ, Brazil; 2 Programa de Pós-graduação em Medicina Tropical, Instituto Oswaldo Cruz (FIOCRUZ), Avenida Brasil 4365, Manguinhos, Rio de Janeiro 21040-360, RJ, Brazil; 3 Instituto Nacional da Mata Atlântica—INMA, Avenida José Ruschi, 4-Centro, Santa Teresa 29650-000, ES, Brazil; 4 Consejo Nacional de Investigaciones Científicas y Técnicas (CONICET), Buenos Aires C1063 CABA, Argentina; 5 Centro Nacional de Diagnóstico e Investigación en Endemo-Epidemias (CeNDIE), Administración Nacional de Laboratorios e Institutos de Salud “Dr. Carlos Malbrán” (ANLIS), Av. Paseo Colón 568, Buenos Aires C1063 CABA, Argentina

**Keywords:** mosquitoes, eggs, wild yellow fever, ovitrap, oviposition height

## Abstract

Culicid species, which include potential vectors of yellow fever, are diverse and abundant, with species commonly co-occurring in certain sites. Studying these species can provide important insights into their vector potential and, consequently, epizootic cycles of arboviruses carried about by these vectors. Here, we evaluated the vertical distribution and temporal segregation of mosquito oviposition with emphasis on arbovirus vectors in a fragment of the Atlantic Forest in Casimiro de Abreu, Rio de Janeiro, Brazil. Two sampling points were selected: Fazenda Três Montes and the Reserva Natural de Propriedade Privada Morro Grande. Collections were carried out at two sites using 10 ovitraps installed on the vegetation cover at different heights (0, 2, 4, 6, and 8 m above ground level) and monitored monthly from July 2018 to December 2020. The hypotheses of temporal and vertical stratification were tested through a PERMANOVA, and the relationship of each species with the vertical distribution was evaluated individually through a correlation analysis. We collected a total of 3075 eggs, including four species of medical importance: *Haemagogus leucocelaenus* (*n* = 1513), *Haemagogus janthinomys* (*n* = 16), *Aedes albopictus* (*n* = 1097), and *Aedes terrens* (*n* = 449). We found that *Hg. leucocelaenus* had a positive relationship with height, exhibiting behavior that appears to benefit from higher heights. The abundance of *Ae. terrens* seemed to follow *Hg. leucocelaenus*, although we did not find a relationship with height for the former species. On the other hand, *Ae. albopictus* exhibited a negative relationship with height, becoming absent or outnumbered at higher strata. Our study site has already presented evidence of recent transmission of the wild yellow fever virus, supporting the need to carefully monitor the emergence of febrile diseases among residents in the surrounding areas and the local population.

## 1. Introduction

From a medical entomology perspective, culicids have significant epidemiological importance due to their potential as a vector of different etiological agents, including arboviruses [1]. Culicidae are widely diverse, distributed worldwide, and have specific habits that influence their zoonotic potential. For example, some species tend to feed more frequently in the treetops, such as *Aedes terrens* Walker, 1956, [2] a habit known as acrodendrophilia. However, recent studies have questioned the acrodendrophilia of these species, given reports of high abundance in the low forest strata [3]. A greater number of vector mosquitoes at the ground level favor the transmission of pathogens between tree-dwelling animals and humans, highlighting the importance of studying oviposition behavior to understand their population distribution and dynamics.

Differences between the breeding sites of different species of Culicidae are probably due to the selection of an oviposition site by the female [4,5], representing an important aspect of the life history of these organisms. In the wild, oviposition sites cover a wide range of available aquatic niches, including on plants, natural breeding grounds caused by the action of wild animals, and artificial breeding grounds, which are water bodies formed as a result of the action of people and domestic animals [6,7]. Differences in oviposition sites can thus ensure the viability of populations and their relative abundance and, consequently, determine their potential as a pathogen vector [8]. For those acrodendrophic species, the larvae breed primarily in tree holes, but many species have also been found in cut or broken bamboo and artificial container sand [9]. An active research program for the study of acrodendrophic culicids has been implemented at the Oswaldo Cruz Institute (Fiocruz) to clarify the ecological aspects of these species and their transmission potential of two specific zoonoses: yellow fever and simian malaria [10].

In Brazil, two genera of culicids stand out as the most important medical sylvatic species: *Aedes* and *Haemagogus*. In this context, *Aedes* is represented by *Aedes albopictus* Skuse, 1894, an invasive species of significant epidemiological importance in the country. It is often synanthropic, transiting between urban, rural, and wild areas. It is considered a sylvatic species with females that exhibit opportunistic oviposition behavior, laying their eggs in both artificial and natural containers. This makes them a possible bridging vector, one that carries pathogens from the wild to the anthropic environment and vice versa [11,12,13]. Together, *Ae. albopictus* and *Ae. terrens* frequently represent the most common sylvatic Aedinis in the Atlantic Forest, both with a wide geographical distribution in Brazil. 

The diverse genus *Haemagogus* includes 28 species, of which nine are found in Brazil [14], some of which are of high epidemiological importance in transmitting the YFV [15]. In the Atlantic Forest region, where our study was carried out, *Ae. albopictus* co-occurs with *Hg. leucocelaenus* and *Haemagogus janthinomys* Dyar, 1921 [8]. However, species of *Haemagogus* show a preference for trunk cavities and, possibly, bamboo holes or fruit rinds [15]. Their eggs are very resistant to drought, commonly hatching at the wettest time of the year, with the eggs of each species exhibiting different behaviors to the stimuli in contact with water, and those of *Hg. janthinomys* may even hatch at the end of the rainy season. Understanding how these species co-exist and co-occur is an important step toward a better understanding of the dynamics of arboviruses in the region. 

Previous studies have extensively proven the vector capacity of *Aedes* and *Heaemogogus* to carry on arboviruses. For instance, Alencar et al. (2021) [10] found the presence of flaviviruses such as the Zika virus (ZIKV) and yellow fever virus (YFV) in *Ae. albopictus* and *Haemagogus leucocelaenus* Dyar and Shannon, 1924, from egg collections, suggesting a possible natural vertical transmission. *Aedes albopictus* is also considered a possible secondary transmitter of other important arboviruses, such as the dengue and West Nile viruses, with its capacity as a vector proven in the laboratory [16,17,18]. Meanwhile, Lourenço-de-Oliveira and Failloux (2017) identified *Ae. terrens* as a potential vector of the chikungunya virus.

Thus, given the medical importance of the species mentioned above, the present study aimed to evaluate the vertical distribution of oviposition and temporal segregation of arbovirus vector mosquitoes in a fragment of the Atlantic Forest of the state of Rio de Janeiro, southeastern Brazil, with a recent severe outbreak of yellow fever (YF). Specifically, we are interested in examining whether it is possible to identify a structuring of the community of mosquito arbovirus vectors based on their vertical stratum and seasonality. In addition, we provide natural history observations about the seasonality and vertical distribution of each species of medical importance at our study site. Thus, our results aim to contribute to a better understanding of vector distribution patterns and provide new insights to better understand epidemiological dynamics.

## 2. Material and Methods

### 2.1. Ethics Statement 

The research was carried out with permission number 44,333 from the Ministry of the Environment (MMA), Chico Mendes Institute for Biodiversity Conservation (ICMBio), and the Biodiversity Information and Authorization System (SISBIO). All team members were vaccinated against the yellow fever virus (YFV) and were aware of the potential risks in the study areas.

### 2.2. Study Site and Data Collection

**Figure 1:**
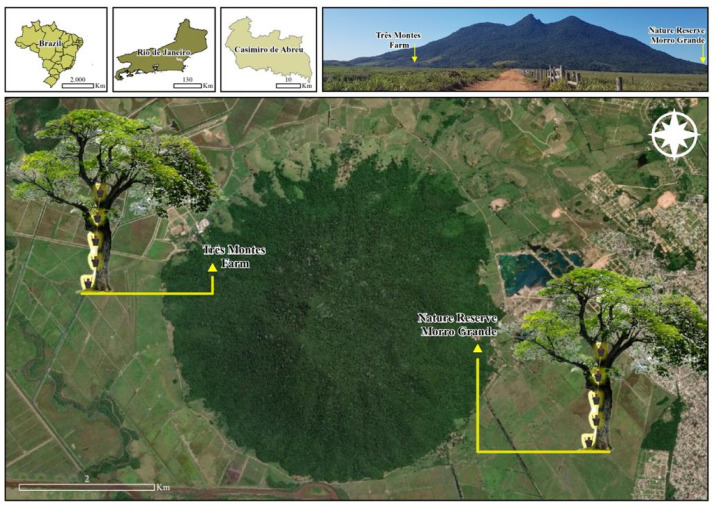
A schematic representation of how ovitraps were hoisted onto the trees is represented along with the location of the two sampling sites at the municipality of Casimiro de Abreu, state of Rio de Janeiro, Brazil: the Fazenda Três Montes (22°31′50.8″ S and 42°02′56.3″ W at an altitude of 314 masl) and the Reserva Particular do Patrimônio Natural Morro Grande (22°32′29.6″ S, 42°00′49.0″ W at an altitude of 314 masl). Image adapted from Google Earth®, Maxar Tecnologies® satellite image, accessed on 8 February 2023.

We selected sampling sites in forested areas near regions with confirmed occurrences of human transmission of YF in the state of Rio de Janeiro, southeastern Brazil. Two fragments of the Atlantic Forest in Casimiro de Abreu were selected for susceptibility to arbovirus transmission. Sampling point 1 (Fazenda Três Montes) was located at 22°31′50.8″ S and 42°02′56.3″ W at an altitude of 314 masl, and sampling point 2 (Reserva Particular do Patrimônio Natural Morro Grande) at 22°32′29.6″ S and 42°00′49.0″ W at an altitude of 314 masl (Figure 1). The region was affected by a recent severe outbreak of YF in 2016–2019 [19]. The two sampling sites are located in the so-called São João River basin, which is defined as an intertropical zone (at low latitudes), and its climate is predominantly tropical [20]. The average climatic condition is represented by a temperature of around 26.8 °C, 56% relative humidity, and an annual precipitation of 1200 mm [20]. In general, the region is highly influenced by the Atlantic Ocean, and the highest levels of precipitation occur from October to March, the months representing most of the spring and summer periods.

From July 2018 to December 2020, the ovitraps were installed at different heights (ground level, 2 m, 4 m, 6 m, and 8 m). Ovitraps were placed on two trees, with one tree per sampling point and one trap at each height. Ovitraps were hoisted onto the tree by throwing a rope weighted with a fishing sinker of approximately 4 cm in diameter and hoisting the trap by a nylon rope up the selected trees, one at each sampling point, for a total of 10 ovitraps, which were sampled monthly. The ovitraps contained plywood sticks, which were numbered sequentially, placed in a damp container, and sent to the Diptera Laboratory, Oswaldo Cruz Institute, FIOCRUZ, Brazil. The ovitraps consisted of an uncovered matte black plastic pot with a capacity of 500 mL, with three 2.5 cm × 14 cm plywood sticks (from Eucatex sheets) fixed vertically inside the trap with clips, following the methodology used by Alencar et al. (2013; 2016) [21,22] and Silva et al. (2019) [23]. Natural water and litter were added to the pot to reproduce a more natural ecosystem.

In the laboratory, the sticks containing eggs were separated, and the eggs were counted and immersed in white polyethylene trays (27 cm × 19 cm × 7 cm) containing dechlorinated water and covered with a screen. Next, the eggs were kept in a controlled experimental environment using an incubator kept at a temperature of 28 °C ± 1 °C, 75–90% relative humidity, and 12 h photoperiod (day/night). They remained in the incubator for three days, with observations performed daily. The larvae were removed from the incubator, placed in beakers, and transferred to breeding cages of 30 × 30 × 30 cm until the emergence of adults.

Adults were identified by direct observation of morphological characteristics under a stereoscopic microscope (Leica DMD108^®^, Wetzlar, Germany) and using dichotomous keys elaborated by Lane (1953), Consoli and Lourenço-de-Oliveira (1994), and Forattini (2002) [7,24,25]. The abbreviations for generic and subgeneric names follow those proposed by Reinert (2009) [26]. After determining the species, all specimens were incorporated into the Entomological Collection of the Oswaldo Cruz Institute, FIOCRUZ, Brazil, under the title “Atlantic Forest—Rio de Janeiro”.

### 2.3. Statistical Analysis 

The influences of seasonality and the vertical stratification on the mosquito assembly were evaluated using a permutational multivariate analysis of variance (PERMANOVA) with 1000 permutations applied to a Bray–Curtis matrix distance with the *adonis2* function in the *vegan* R package [27,28]. The abundance of eggs was log+1 transformed prior to analysis to reduce the influence of the dominant rate on dissimilarity patterns. Our analyses were stratified by site, and the marginal effects of each variable were compared by their R^2^ and F-statistics [28].

## 3. Results

We collected a total of 3075 culicid eggs. Of this total, those that survived to adulthood belonged to the following species of medical importance: *Hg. leucocelaenus* (*n* = 1513), *Hg. janthinomys* (*n* = 16), *Ae. albopictus* (*n* = 1097), and *Ae. terrens* (*n* = 449). Our PERMANOVA model was able to capture 82.99% of the total variation in the abundance matrix (R^2^ = 0.834), with height (R^2^ = 0.464, $F_{Height}$ = 9.833, *p* < 0.001) and season (R^2^ = 0.228, $F_{Season}$ = 6.436, *p* < 0.001) explaining a significant degree of the mosquito species composition at the studied site. 

### Vertical Distribution of Vector Mosquitoes and Spatiotemporal Segregation

**Figure 2:**
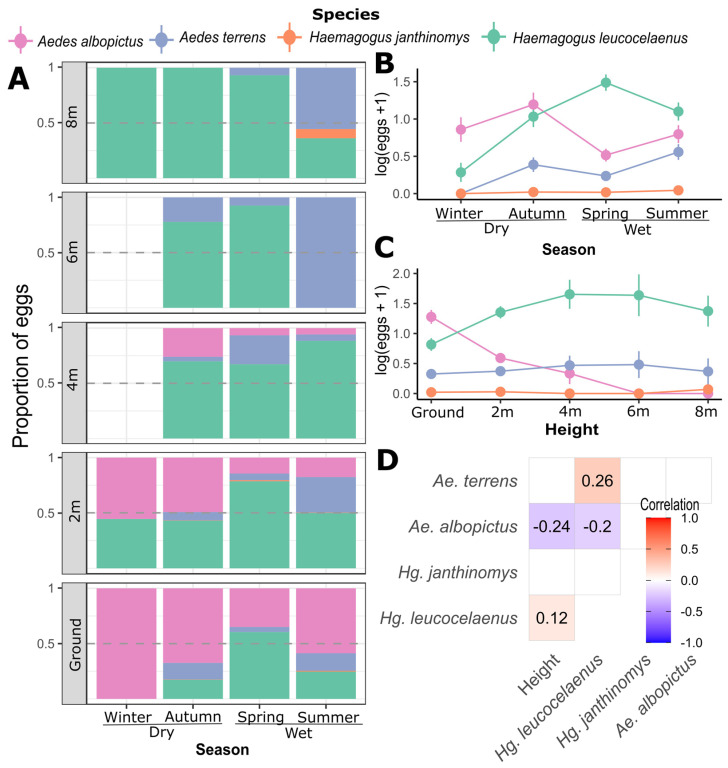
(**A**) Records of eggs (measured as a proportion of the total) are plotted against season and height (0 m to 8 m). On the right side, log-transformed egg counts are presented against season (**B**) and height (**C**). Vertical bars represent standard error. (**D**) A correlation matrix shows simple pairwise Pearson correlations between species and height and between pairs of species, which are shaded red or blue when significant (*p* < 0.05). Winter and autumn represent the driest seasons, and spring and summer the wettest seasons at the study site.

The vertical distribution of the species at the two sampling points shows that *Hg. leucocelaenus* was present at all heights above the ground where the ovitraps were distributed, presenting greater abundance in the ovitraps installed at the level of 4 to 6 m. Although *Ae. terrens* had a slight increment in abundance over intermediate heights, we did not find a significant correlation with height (*p* = 0.98), despite being significantly associated with the abundance of *Hg. leucocelaenus* (*p* < 0.001; Figure 2D). Unlike *Hg. leucocelaenus*, *Ae. albopictus* was more abundant in ovitraps at ground level and completely absent in the ovitraps located at 6 m and 8 m above the ground, exhibiting a negative relationship between abundance and height (*p* < 0.001; Figure 2C). The eggs of *Hg. janthinomys* were present in the greatest numbers at 8 m and were less common at the ground level and 2 m; moreover, they were absent at the intermediate height.

**Figure 3:**
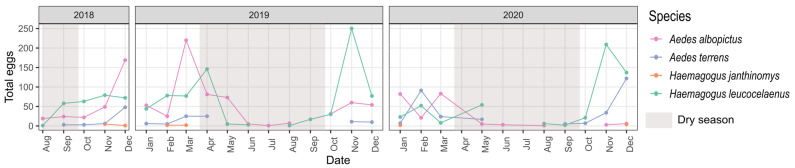
Monthly distribution of Culicidae species at sample points from August 2018 to December 2020, municipality of Casimiro de Abreu, state of Rio de Janeiro, Brazil.

Seasonality was also an important factor affecting the species composition of the sampled eggs (Table 1, $F_{Season}$ = 6.436 *p* < 0.001). Examining the egg counts for each month, *Ae. albopictus* and *Hg. leucocelaenus* were the most abundant species throughout the year, followed by *Ae. terrens* and *Hg. janthinomys* (Figure 3). The winter season had the lowest overall number of eggs (Figure 2B). *Aedes terrens* and *Hg. janthinomys* were the species to disappear earlier during the driest seasons, while *Hg. leucocelaenus* and *Ae. albopictus* were present during almost the entire driest seasons (Figure 3). Despite these differences, we detected a similar trend for all species in increasing the abundance of eggs toward the wet seasons and decreasing the abundance during the dry seasons (Figure 3). We only found a significant correlation between *Hg. leucocelaenus* and *Ae. terrens* (Figure 2D). The vertical stratification among the species likely exerts a greater influence on them, masking such temporal correlation. This result is also supported by a greater contribution from height than by season, as indicated by the PERMANOVA ($F_{Height}$ > $F_{Season},\;$Table 1).

Figure 2 shows the proportion of records of each species throughout the seasons and height unveiling important patterns (see Figure 2A). For example, *Ae. albopictus* was dominant at the ground level but quickly yielded to *Hg. leucolcelaenus* in the higher strata. While *Ae. albopictus* was the most common species at the ground level throughout the year, it was challenged by *Hg. leucocelaenus* in the higher strata. At the ground level, *Ae. albopictus* was dominant in winter, whereas *Hg. leucocelaenus* gained prominence until spring. Each species tended to make up about 50% of the total eggs in the ovitraps, marking a low point in the proportional dominance of *Ae. albopictus* at ground level. In autumn, spring, and summer, *Hg. leucocelaenus* was the dominant species at 2 m and higher strata. Throughout the sampling period, *Ae. albopictus* was the dominant species at soil height and *Hg. leucocelaenus* was the most abundant at the higher vertical strata. At site-specific data, eggs of *Ae. terrens* were more abundant around ovitraps at 4 m in the Fazenda Três Montes. However, we neither found a significant relationship with *Ae. terrens* and height in any sampling site nor a similar trend at Campo Grande (see Supplementary Materials Figures S2 and S3). 

## 4. Discussion

The study of the vertical distribution of oviposition and temporal segregation of mosquitoes in a fragment of the Atlantic Forest in Rio de Janeiro allowed us to observe four epidemiologically important species: *Hg. leucocelaenus*, *Hg. janthinomys*, *Ae. albopictus*, and *Ae. terrens*. This allowed us to gain insights into the transmission of pathogens between tree-dwelling animals and humans based on the vector species found at different elevations. This is particularly important given the recent outbreak of yellow fever in the region in 2016–2019 [29].

Both genera (*Haemagogus* and *Aedes*) were found in all seasons, highlighting the medical importance of these taxa. In the present study, *Hg. leucocelaenus* and *Ae. albopictus* were the most abundant, with the former present in greater abundance throughout the year in most strata. Beier et al. (1983) [30] found that several species can cohabit, as noted in the eggs collected from the ovitraps at soil level and 2 m; however, only one or two predominated in their study. This is consistent with our findings of *Hg. leucocelaenus* eggs, which co-occurred with two or three species on the same stick.

The richness of mosquitoes was higher in the samples collected in the ovitraps located at ground level and lower at higher strata, which might be related to the decreased availability of hosts at higher strata. Our findings corroborate observations made by Alencar et al. (2016) [22], who reported that *Hg. janthinomys*, even with a quantitatively smaller population relative to *Hg. leucocelaenus*, frequented ovitraps located at the highest level of the canopy. Despite being assigned as acrodendrophilic mosquitoes, *Haemagogus* specimens were found at different heights during the study. For instance, despite being more frequent at higher strata, *Hg. leucocelaenus* was also present at all heights in both locations, while *Hg. janthinomys* was almost absent in the locality of Fazenda Três Montes and present at two heights in Morro Grande. According to Alencar et al. (2013) [21], *Hg. leucocelaenus* demonstrated similar behavior since it was reported in all ovitraps installed at different heights. These results suggest that *Haemagogus* species may exhibit a plastic-specific vertical distribution behavior. This plastic mobility is likely to reflect their generalist dietary habits. Alencar et al. (2008) [31] reported the dietary habits of *Hg. leucocelaenus* and *Hg. janthinomys*, considering them generalist species in terms of their dietary habits. This may help explain the higher abundance of the species throughout the year and at both sites. Alencar et al. (2018) [32] confirmed the species’ generalist feeding habits as well as their mobility between the soil and the canopy in search of a possible food source. 

Among the *Aedes* species detected, *Ae. terrens* was found at all heights at the sampling point of Fazenda Três Montes; this result corroborates an observation made by Alencar et al. (2013) [21] who found the presence of *Ae. terrens* at all heights, except in ovitraps at a height of 1.80 m, and noted their tendency to spawn in ovitraps between the heights of 2.50 and 4.30 m. It should be noted that the highest trap in their study was located at 4.30 m. The epidemiological importance of *Ae. terrens* lies in the ability to transmit the chikungunya virus in an experimental trial, according to Lourenço-de-Oliveira and Anna-Bella Failloux (2017) [33]. On the other hand, individuals of *Ae. albopictus*, a potential vector of flavivirus, showed a marked preference for lower strata and was most abundant at ground level at both sample points, consistent with the observations of Alencar et al. (2013) [21]. The *Ae. albopictus* has also been reported as a natural vector of ZIKV in several countries [11,34,35], raising concerns about a wild cycle for ZIKV in South America since this species inhabits forests and peridomestic environments in Rio de Janeiro [36]. 

The oviposition pattern of the species found in the present study may be associated with intra- or hetero-specific competition. Therefore, it demonstrates the importance of performing other evaluations on the oviposition behavior of females as it relates to vertical strata. The Atlantic Forest fragment studied has already shown evidence of recent transmission of the wild YFV. Due to the strong presence of the main mosquito vectors in Brazil, we believe that special attention should be given to monitoring the emergence of febrile diseases among residents in the surroundings and the local population.

## 5. Conclusions

We investigated the spatial and temporal distribution of oviposition by culicids with significant epidemiological importance due to their potential as a vector of different etiological agents, including arboviruses. We found evidence to support the temporal and spatial segregation of a community comprising four species of culicids in a region with a recent outbreak of yellow fever virus (YFV). This spatiotemporal variation in the oviposition by culicids in our study site supports the acrodendrophilic behavior assumed by *Hg. leucocelaenus*; however, the presence of such behavior was inconclusive for *Ae. terrens* and *Hg. janthinomys*. On the other hand, *Ae*. *albopictus* was associated with lower heights. Finally, given the potential vector for arboviruses of these species and the historical records of YFV in the study site, our results provide important information on the epidemiologic dynamics of arborvirus outbreaks in the region. Further studies might be particularly useful to refine the importance of species-specific behavior to determine the species composition in the vertical strata of the forests for sylvatic culicids.

## Figures and Tables

**Table 1:** Permutational multivariate analysis of variance (PERMANOVA) summary table showing degrees of freedom (df), the sum of squares, R^2^, F-statistics, and respective *p*-values.

	df	Sum of Squares	R^2^	F	*p*-Value
Height	4	2.394	0.464	9.833	<0.001
Season	3	1.175	0.228	6.436	<0.001
Height × Season	10	0.735	0.142	1.207	0.256
Residuals	14	0.852	0.165		
Total	31	5.155	1.000		

## Data Availability

Data and script are available under request.

## Supplementary Materials

The following supporting information can be downloaded at: https://www.mdpi.com/article/10.3390/tropicalmed8050256/s1. Table S1. Permutational Multivariate Analysis of Variance (PERMANOVA) summary table, showing degrees of freedom (df), sum of squares, R², F-statistics, and respective *p*-values; Figure S1. Egg records (measured as a proportion of the total) plotted against season and height (ground level = 0 m to 8 m) for each sampling site, along with the polled data. The pooled data are presented in Figure 2A in the main manuscript; Figure S2. Log-transformed egg counts presented against season and height. The pooled data are represented in the main manuscript by Figure 2B,C for season and height, respectively; Figure S3. Matrices with simple pairwise Pearson correlations between species and height and between pairs of species for each site and pooled data. Shaded red or blue cells represent significant (*p* < 0.05) correlations. The correlation matrix for the pooled data is informed in the main manuscript (Figure 2D); Figure S4. Monthly distribution of Culicidae species at each sampling site and pooled data from August 2018 to December 2020, Casimiro de Abreu, Rio de Janeiro, Brazil. The pooled data are informed in the main manuscript as Figure 3.
