# Supplementary material for: Vertical Distribution of Oviposition and Temporal Segregation of Arbovirus Vector Mosquitoes (Diptera: Culicidae) in a Fragment of the Atlantic Forest, State of Rio de Janeiro, Brazil
Source: Trop Med Infect Dis. 2023 Apr 29;8(5):256. doi: 10.3390/tropicalmed8050256 (PMC10221014; doi:10.3390/tropicalmed8050256)
Supplement: Supplementary file 1 [file tropicalmed-08-00256-s001.zip › tropicalmed-2313869-supplementary.pdf]

Tropical Medicine and Infectious Disease

MDPI

Supplementary Materials of Vertical Distribution of Oviposition and Temporal Segregation of Arbovirus Vector Mosquitoes (Diptera: Culicidae) in a Fragment of the Atlantic Forest, State of Rio de Janeiro, Brazil

Site-specific analyses are compared with pooled data presented in Dias et al. 2023 in *Trop. Med. Infect. Dis. Journal*. Methods are described in detail in the main manuscript. The pooled data represent the sum of the total egg records in two sampling sites grouped by seasons and heights. For this reason, we could not perform the Permutational Multivariate Analysis of Variance (PERMANOVA) with the interaction between terms for site-specific analyses. Nevertheless, in general, the same patterns found using pooled data were found in site-level analyses. In particular, PERMANOVA showed a very similar result for both sites (Table S1). When egg records are plotted against seasons and height for each site, we observed interesting findings emerging along expected nuances, raising the importance of further studies on the topic. For example, the pairwise correlation for pooled or site-specific analyses supports a strong negative relationship between *Aedes albopictus* and height (Figure S4). On the other hand, *Hg. leucoceleanus* and height were positively and significantly correlated with height in the Reserva Particular do Patrimônio Natural Morro Grande but not in the Fazenda Três Montes (Figure S4). Meanwhile, the egg abundance of *Hg. leucoceleanus* at 8m was higher than at the ground level, and intermediate heights were highly variable, which might explain the lack of a significant result (Figure S3). Such a finding highlights the highly variable response of these organisms that are rapidly affected by microhabitat availability and the environmental changes that justify the importance of multiple studies to clarify the variation in the species' behavior along their distribution range and their site-specific importance as a vector of arboviruses.

Table S1. Permutational Multivariate Analysis of Variance (PERMANOVA) summary table, showing degrees of freedom (df), sum of squares, R^2, F-statistics, and respective *p*-values.

	**df**	**Sum of Squares**	**R^2**	**F**	***p*-value**
			**Pooled**		
Height	4	2.393	0.464	9.833	< 0.001
Season	3	1.175	0.228	6.436	< 0.001
Height × Season	10	0.7346	0.142	1.207	0.271
Residual	14	0.8519	0.165		
Total	31	5.155	1.000		
			**Fazenda Três Montes**		
Height	4	1.586	0.513	6.201	0.001
Season	3	0.868	0.280	4.524	0.002
Residual	10	0.639	0.207		
Total	17	3.093	1.000		
			**Reserva Particular do Patrimônio Natural Morro Grande**		
Height	4	1.005	0.594	5.588	0.003
Season	3	0.415	0.245	3.073	0.040
Residual	6	0.270	0.160		
Total	13	1.690	1.000		

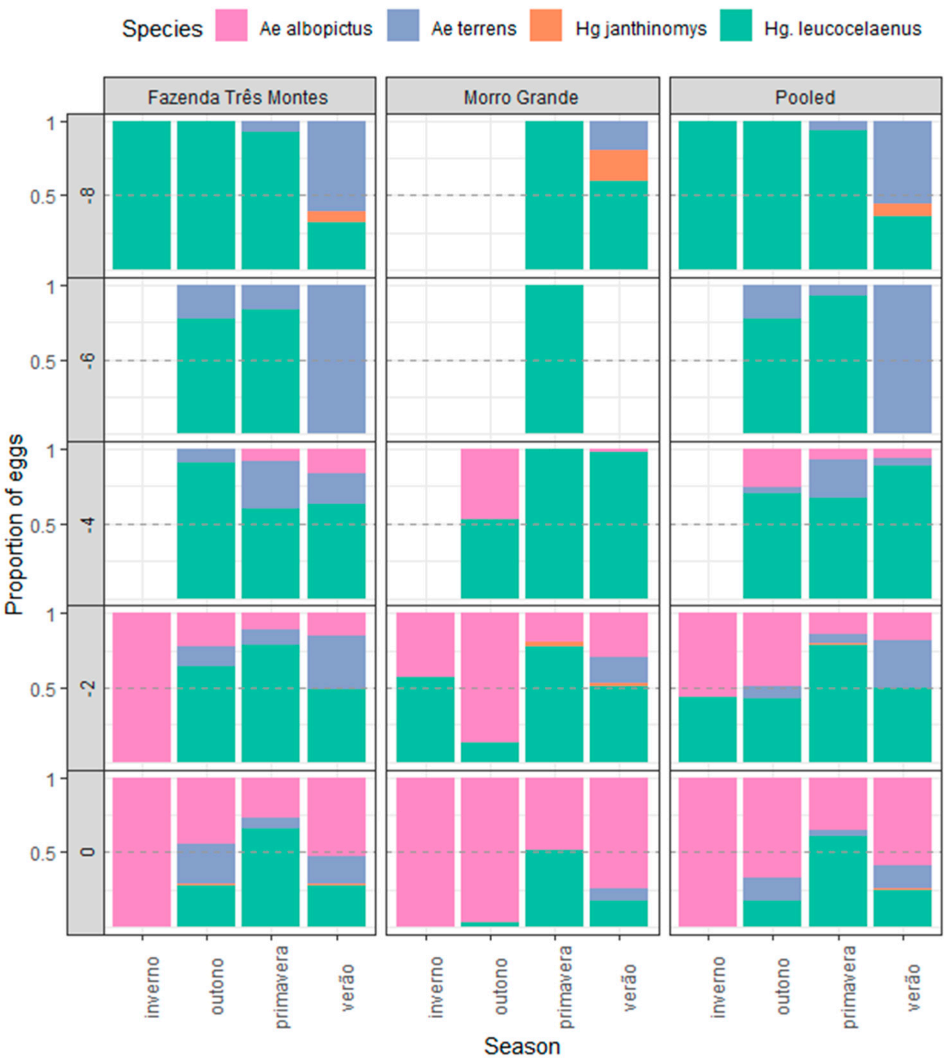

Figure S1. Egg records (measured as a proportion of the total) plotted against season and height (ground level = 0 m to 8 m) for each sampling site, along with the polled data. The pooled data are presented in Figure 2A in the main manuscript.

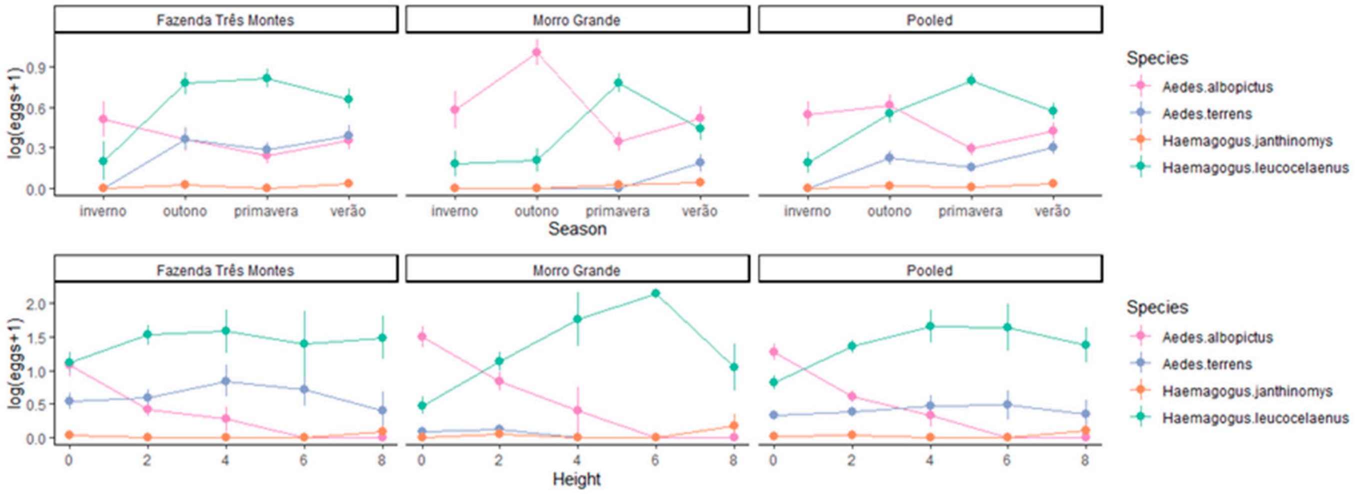

Figure S2. Log-transformed egg counts presented against season and height. The pooled data are represented in the main manuscript by Figures 2B and 2C for season and height, respectively.

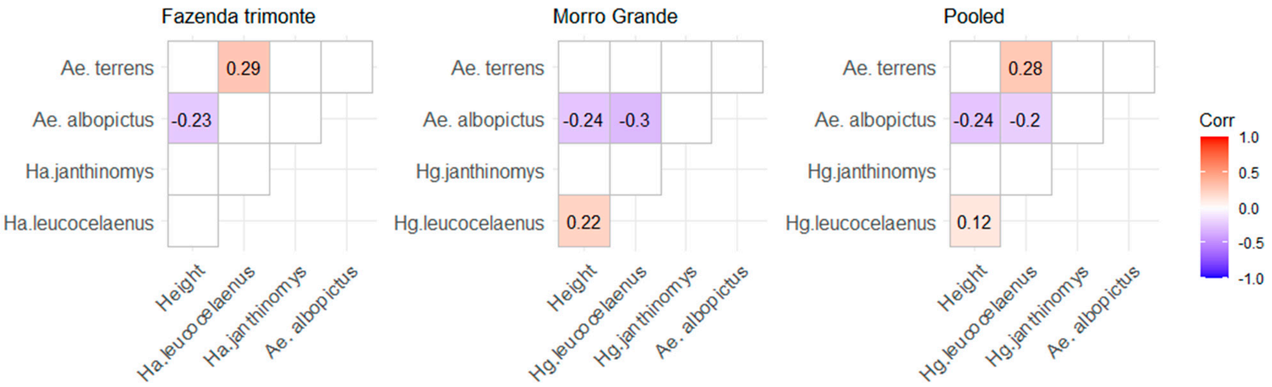

Figure S3. Matrices with simple pairwise Pearson correlations between species and height and between pairs of species for each site and pooled data. Shaded red or blue cells represent significant ($p < 0.05$) correlations. The correlation matrix for the pooled data is informed in the main manuscript (Figure 2D).

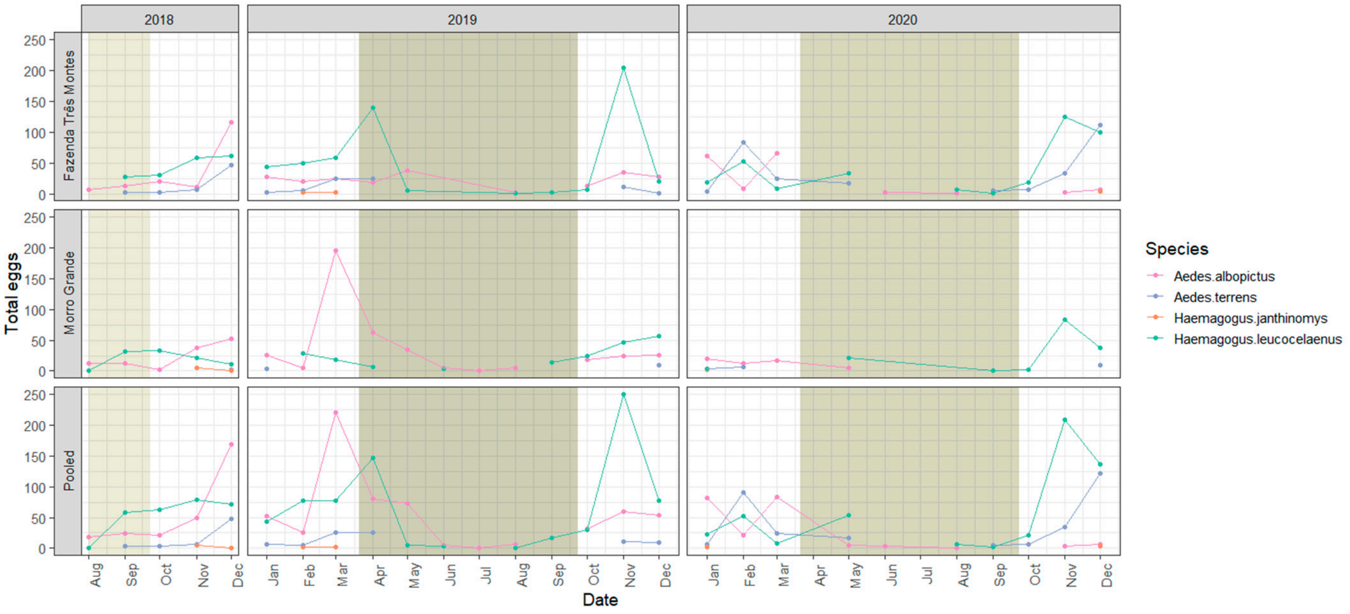

Figure S4. Monthly distribution of Culicidae species at each sampling site and pooled data from August 2018 to December 2020, Casimiro de Abreu, Rio de Janeiro, Brazil. The pooled data are informed in the main manuscript as Figure 3.
